# Effects of increasing cold exposure on the oxygen uptake of walking unloaded and loaded

**DOI:** 10.1186/2046-7648-4-S1-A56

**Published:** 2015-09-14

**Authors:** Katrina Hinde, Carlton Cooke, Ray Lloyd

**Affiliations:** 1 School of Sport, Carnegie Faculty, Leeds Beckett University, Leeds, UK; 2 Leeds Trinity University, Leeds, UK

## Introduction

Cold exposure and load carriage is an understudied area. Most research shows that VO_2max _is generally unaffected by cold exposure, however the majority of research suggests that sub-maximal O_2 _consumption increases for a given workload [1]. This pilot study assessed the effects of cold on load carriage.

## Methods

4 male participants (age: 21.8 ± 3.4 years, height: 182.5 ±5.0 cm, weight: 77.8 ± 13.5 kg) completed a walking protocol of ~1 hour in a range of different ambient temperatures within an environmental chamber (20 °C, 10 °C, 5 °C, 0 °C, -5 °C and -10 °C). Humidity was controlled at ~50% while altitude was 0 m (20.95% FiO_2_). Participants wore shorts and t-shirt for all trials. The protocol included a 15 minute rest period, unloaded walking at 4 km.hr^-1 ^for 4 minutes at 0% and 10% gradient. The same workloads were repeated loaded (18 kg) after a 5 minute rest. Heart rate returned to resting levels before each exercise section to ensure prior activity did not influence findings. Unloaded walking was then repeated. Expired air was collected and analysed using a Cortex 3B Metalyzer (Germany). Statistical analysis was performed using SPSS version 22, with significance denoted by p < 0.05.

## Results

Table 1 shows a significant increase in VO_2 _with load (p = 0.019). At all workloads, significant increases in VO_2 _were associated with decreasing temperature (p = 0.048). ΔVO_2 _values suggest that the effect of loading was consistent, regardless of ambient temperature (p = 0.997). When comparing the first unloaded exercise bout with the second, VO_2 _for 20 °C, 10 °C and 5 °C was similar, whereas at 0 °C and below, VO_2 _was higher in the second unloaded bout, but this interaction was not significant (p = 0.158).

**Table 1:** Mean ± SD VO_2 _responses (ml.kg^-^^1^.min^-^^1^) averaged across 0% and 10% gradient

	20 °C	10 °C	5 °C	0 °C	-5 °C	-10 °C
Unloaded 1	18.69 ± 1.43	18.99 ± 1.52	16.84 ± 4.42	19.30 ± 2.38	22.16 ± 1.50	22.99 ± 1.09

Loaded	21.66 ± 2.33	23.76 ± 0.41	20.41 ± 5.99	24.43 ± 4.06	24.68 ± 1.64	27.44 ± 4.13

ΔVO_2 _	2.98 ± 1.55	4.78 ± 1.26	3.58 ± 2.06	5.13 ± 3.84	2.51 ± 2.71	4.45 ± 4.55

Unloaded 2	18.73 ± 1.52	19.03 ± 0.66	17.89 ± 5.73	23.41 ± 7.72	29.15 ± 5.91	28.25 ± 5.53

## Discussion

The effect of ambient temperature on loading was not significant, however a decrease in temperature generally increased oxygen uptake. Reasons for a higher VO_2 _response during cold exposure could be due to shivering in an attempt to maintain core temperature [2]. However, the exercise intensity was above the estimated 1.5 L.min^-1 ^threshold for the shivering response, therefore it is unlikely that this was the sole reason [3]. VO_2 _can be increased by non-shivering thermogenesis [4], this is heat production from sources excluding muscle contraction and involves calorigenic hormones and brown fat metabolism. Muscle strength has also been seen to decrease in cold environments through a decrease in contractile force [1,5]. More motor units are therefore recruited to meet the exercise demands, thus increasing VO_2._
